# Imposter syndrome as mediator and moderator between personality and mental health in Malaysian students

**DOI:** 10.1038/s41598-026-46843-w

**Published:** 2026-04-03

**Authors:** Nafiseh Kananifar, Danilo Garcia

**Affiliations:** 1 Department of Psychology, University of Stavanger, Stavanger, Norway; 2 Department of Psychology, Promotion of Health and Innovation for Well-Being (PHI-WELL), University of Stavanger, Stavanger, Norway; 3 Department of Behavioral Sciences and Learning, Linköping University, Linköping, Sweden; 4 Lab for Biopsychosocial Personality Research (BPS-PR), International Network for Well-Being, Stavanger, Norway; 5 Promotion of Health and Innovation (PHI) Lab, International Network for Well-Being, Linköping, Sweden; 6 Department of Psychology, University of Gothenburg, Gothenburg, Sweden; 7 Centre for Ethics, Law and Mental Health (CELAM), University of Gothenburg, Gothenburg, Sweden

**Keywords:** Imposter Syndrome, Personality Traits, Mental Health, Mediation, Moderation, University Students, Health care, Psychology, Psychology

## Abstract

We explored whether Imposter Syndrome functions as a mediator or a moderator in the relationship between personality traits and mental health outcomes among university students in Malaysia. A total of 755 students completed validated measures of personality (IPIP-NEO), Imposter Syndrome (CIPS), and mental health (GHQ-28). We used structural equation modeling (SEM) to estimate bootstrapped indirect effects for mediation and tested moderation by including personality x Imposter Syndrome interaction terms in SEM. Imposter Syndrome showed small but significant indirect effects linking both Neuroticism (*β* = 0.102, *p* < 0.001) and Agreeableness (*β*= -0.024, *p* = 0.014) to mental health problems. In the moderation model, the Conscientiousness x Imposter Syndrome interaction (*β* = 0.07, *p* < 0.001) and the Neuroticism x Imposter Syndrome interaction (*β* = − 0.04, *p* < 0.001) were statistically significant but small in predicting mental health problems. These findings indicate that although Imposter Syndrome showed statistically detectable mediating and moderating associations for some personality traits, the effects were small and likely of limited practical significance. Overall, Imposter Syndrome did not consistently function as a robust mechanism (mediation) or boundary condition (moderation) across the Big Five traits. Future research should identify mechanisms beyond impostor feelings (e.g., self-regulatory capacities) that may better explain the link between personality traits and mental health.

## Introduction

Mental health challenges among university students are rising worldwide, including in East and Southeast Asia, such as Malaysia. Stress, anxiety, depression, and social dysfunction are increasingly reported by students as they navigate academic, social, and personal demands^1–5^. These challenges are intensified by cultural and societal expectations, financial burdens, and the rapid transition into adulthood^6–8^. In order to understand the underlying determinants, researchers have turned their attention to individual differences as vulnerability factors. Among which, personality traits^9–11^ and Imposter Syndrome^12,13^ stand out.

Indeed, personality, including the Big Five traits (Openness, Conscientiousness, Extraversion, Agreeableness, and Neuroticism), is consistently linked to mental health^14,15^. For instance, Neuroticism is often associated with heightened emotional reactivity and increased susceptibility to anxiety^16,17^ and depression^18,19^, while traits like Conscientiousness and Agreeableness are commonly associated with adaptive functioning^20,21^. Imposter Syndrome, defined by self-doubt, feelings of intellectual and performance fraudulence, and an inability to internalize success^22^, has independently also been linked to poor mental health outcomes such as anxiety and depression^23,24^. Some studies, for example, have shown that Imposter Syndrome not only correlates with Neuroticism but also intensifies feelings of inadequacy, anxiety, and depression, highlighting its role as a psychological risk factor in academic and professional contexts^25,26^.

Nevertheless, research on the psychological mechanisms through which Imposter Syndrome operates in relation to personality and students’ mental health show mixed results. One study, for example, could not verify Imposter Syndrome as a mediator between maladaptive perfectionism and depression^27^, whereas another study reported a full mediation by Imposter Syndrome as the link between perfectionism and anxiety and partially mediating perfectionism’s association with depression^28^. Moreover, using Imposter Syndrome as a moderator, researchers have shown that the positive link between perfectionism and depression disappears at low imposter feelings, indicating that Imposter Syndrome can act as a boundary condition^28^. Additional studies among university students support a mediating role of Imposter Syndrome, linking maladaptive perfectionism to suicidal ideation^29^, connecting perfectionism to lower happiness^30^, and, over six months and together with self-compassion, mediating the effects of perfectionism to later depressive symptoms^31^. Collectively, these findings underscore the complex direct and indirect role of Imposter Syndrome in mental health. In other words, suggesting that Imposter Syndrome can simultaneously operate as a mechanism and as a contextual factor shaping mental health outcomes.

To the best of our knowledge, in relation to personality traits, researchers predominantly treat Imposter Syndrome as a direct correlate of poorer mental health or, less commonly, as a mediator or a moderator^32,33^. Notably, tests of mediation and moderation in the personality-mental health link have emphasized perfectionism rather that the Big Five traits, and as detailed earlier, the evidence remains inconsistent. Some few studies have examined the dual roles of Imposter Syndrome in other domains. Cokley and colleagues (2017), for example, showed that imposter feelings mediated the effect of perceived discrimination on anxiety and depression and moderated the discrimination-mental health relationship, with effects varying across different ethnic groups. Taken together, prior findings underscore the need to test, within a single framework, whether Imposter Syndrome operates as a mechanism (i.e., mediation) or a contextual amplifier (i.e., moderation) of the Big Five–mental health associations.

The distinction between mediation and moderation is both of methodological and practical importance, bridging theoretical insight and real-world application^34^. Methodologically, if Imposter Syndrome functions as a mediator, personality traits influence mental health indirectly through imposter-related cognitions, thus identifying a psychological mechanism. If, instead, Imposter Syndrome acts as a moderator, the strength or direction of the personality-mental health association depends on how strongly individuals endorse imposter feelings, thus specifying a contextual role. This distinction also guides intervention. If Imposter Syndrome mediates trait effects (e.g., Neuroticism → imposter self-doubt → distress), interventions should target the mechanism (e.g., challenge maladaptive self-beliefs, bolster self-efficacy). If it moderates trait effects, support should be selectively tailored to students high in imposter feelings, equipping them with skills to buffer the impact of vulnerable trait profiles. Such precision is especially relevant in high-pressure academic settings where evaluation and competition are salient.

Theoretically, both roles are plausible. Trait Activation Theory proposes that situational cues (e.g., evaluation, competition, high standards) activate underlying dispositions, eliciting self-critical appraisals that are characteristic of individual who score high in Imposter Syndrome^35^. In this view, imposter cognitions can operate as a mechanism linking traits (e.g., high Neuroticism, perfectionistic concerns) to anxiety and depression. Person-Environment Fit models, in turn, emphasize the match/mismatch between dispositions and contextual demands^36,37^. Imposter feelings might condition when and for whom trait liabilities translate into poorer mental health, which is consistent with evidence that the perfectionism-depression link weakens or disappears when imposter tendencies are low^28^. Ergo, these perspectives support testing Imposter Syndrome as both a mediator (mechanism) and a moderator (contextual amplifier) of the Big Five-mental health associations—an approach that directly addresses the mixed findings in the literature and guides the design of more precise, targeted interventions.

### The Present Study

Although Imposter Syndrome is widely discussed as a negative correlate of mental health, few studies have evaluated its mediating and moderating roles side by side using comparable models. Moreover, Malaysia’s academic context, characterized by high performance expectations and collectivistic norms, offers a relevant setting for understanding how personality and self-doubt interact to influence mental health^38–40^. Using previously collected data^41^, we estimated two complementary models, (a) a mediation model testing indirect effects via Imposter Syndrome and (b) a set of moderation models testing latent interactions between each trait and Imposter Syndrome. The Big Five served as predictors, Imposter Syndrome as mediator/moderator, and a latent mental health factor as the outcome. This design allows a direct empirical comparison of mechanism (mediation) versus contingency (moderation) while holding the measurement model and estimation approach constant. Consistent with prior work, we anticipated a prominent role for Neuroticism, directly and indirectly via Imposter Syndrome, with other traits examined more exploratorily.

## Method

### Ethical statement

This study was approved by the Medical Ethics Committee at the University Malaya Medical Centre (Reference Number: 1010.74). Participation was voluntary, anonymous, and written informed consent was obtained from all participants following ethical guidelines established by the Declaration of Helsinki and Malaysian regulations for research involving human subjects.

### Participants and procedure

The study included 755 university students from the University of Malaya, including 352 international students (205 males, 147 females) and 403 Malaysian students (170 males, 233 females). Participants ranged in age from 19 to 54 years (*M* = 25.88, *SD* = 6.27) and represented both undergraduate and postgraduate programs. The sample was obtained through convenience sampling during the first week of the academic semester from lecture halls and common areas during working hours on campus. Demographic information, including age, gender, and nationality, was collected alongside the questionnaires^41^.

### Measures

#### Personality

The NEO Five Factor Inventory^42^ was used to measure the Big Five personality traits: Openness (e.g., “I don’t like to waste my time daydreaming.”), Conscientiousness (e.g., “I keep my belongings neat and clean.”), Extraversion (e.g., “I like to have a lot of people around me.”), Agreeableness (e.g., “I would rather cooperate with others than compete with them.”), and Neuroticism (e.g., “I am not a worrier.”). This validated 60-item scale uses a 5-point Likert scale (1 = *strongly disagree*, 5 = *strongly agree*) and demonstrated strong internal consistency across subscales in the present study: Cronbach’s alpha for Openness = 0.88, for Conscientiousness = 0.92, for Extraversion = 0.92, for Agreeableness = 0.91, and for Neuroticism = 0.92.

#### Imposter syndrome

The Clance Imposter Phenomenon Scale^43^ was employed to assess Imposter Syndrome. This 20-item instrument uses a 5-point Likert scale (1 = *not at all true*, 5 = *Very true*) to measure self-doubt, fear of exposure, and the tendency to discount accomplishments. Example of items are: “I avoid evaluations if possible and have a dread of others evaluating me.”, “I sometimes think I obtained my present position or gained my present success because I happened to be in the right place at the right time or knew the right people.”, “If I receive a great deal of praise and recognition for something I’ve accomplished, I tend to discount the importance of what I’ve done”. Cronbach’s alpha in the present study was 0.91.

#### Mental health problems

The General Health Questionnaire 28^44^ items was used to assess mental health outcomes, focusing on four subscales: Anxiety (e.g., “Have you recently been getting scared or panicky for no good reason?”), Depression (e.g., “Have you recently been thinking of yourself as a worthless person?”), Somatization (e.g., “Have you recently been feeling perfectly well and in good health?”; reversed item), and Social Dysfunction (e.g., “Have you recently been taking longer over the things you do?”). This 28-item scale employs a 4-point Likert scale (1 = *Better than usual*, 4 = *Much worse than usual*). Cronbach’s alphas in the present study were 0.89 for Anxiety, 0.92 for Depression, 0.89 for Social Dysfunction, 0.88 for Somatization, and 0.93 for the total score (i.e., Mental Health Problems).

### Statistical procedure

**Fig. 1:**
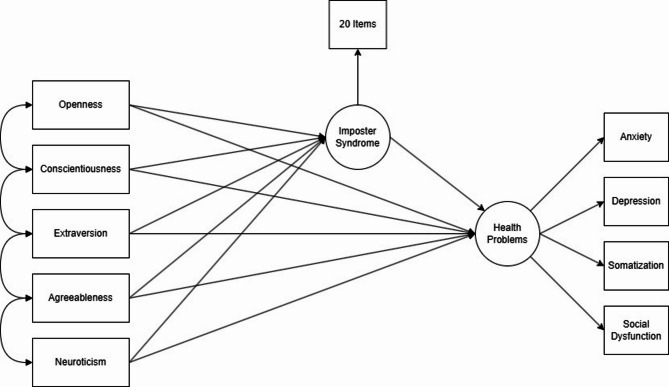
Conceptual Mediation Model: Big Five → Imposter Syndrome → Mental Health. Note. The model specifies direct paths from each Big Five trait (Openness, Conscientiousness, Extraversion, Agreeableness, Neuroticism) to Imposter Syndrome and to Mental Health, plus the mediating path from Imposter Syndrome to Mental Health Problems.

**Fig. 2:**
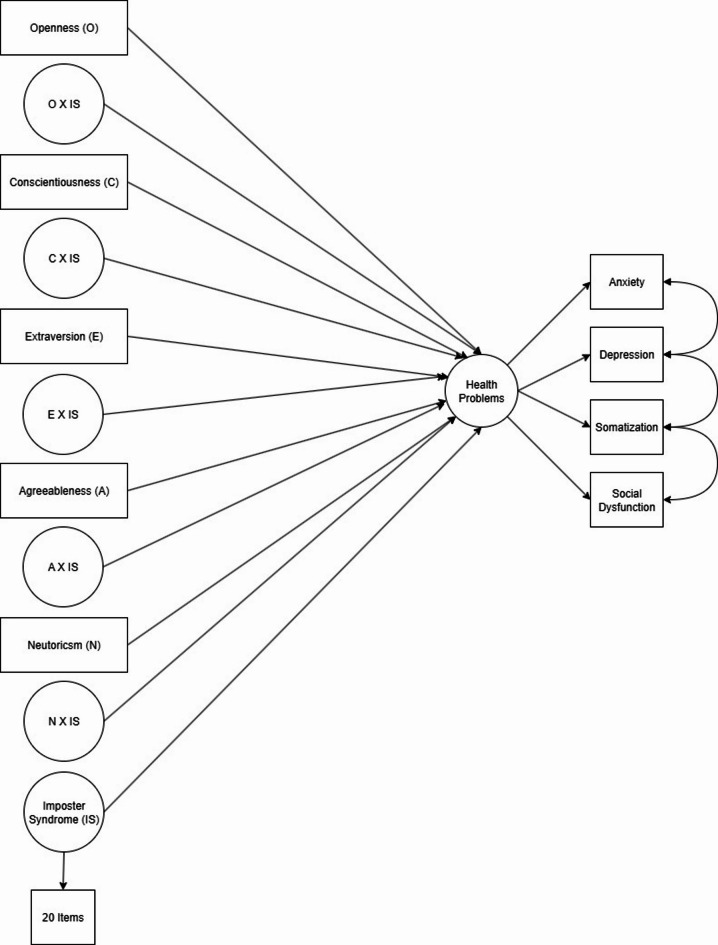
Conceptual Moderation model: imposter syndrome conditions big five trait–mental health associations. Note. The model includes main effects of each Big Five trait and Imposter Syndrome (IS) on Mental Health, plus an interaction term for each trait × IS.

All analyses were conducted using R. Descriptive, mediation, and moderation analyses were performed using the lavaan package, which facilitates comprehensive Structural Equation Modeling (SEM). SEM was used to examine the overall model fit and test relationships among variables within the hypothesized framework. Model adequacy was evaluated using widely accepted SEM fit indices, such as Comparative Fit Index (CFI > 0.90), Root Mean Square Error of Approximation (RMSEA < 0.08), and Standardized Root Mean Square Residual (SRMR < 0.08). To test mediation effects, we applied bootstrapping with 5,000 resamples to deal with any potential multivariate normality issues, thus, ensuring robust inference and reliable estimation of mediation pathways. Trait-specific mediation pathways (e.g., Neuroticism → Imposter Syndrome → Health) were analyzed to identify the unique contributions of each personality trait (predictor) to mental health outcomes through Imposter Syndrome (mediator) (see Fig. 1). Moderation effects were tested by creating interaction terms between each personality trait (predictors) and Imposter Syndrome (moderator) (e.g., Neuroticism × Imposter Syndrome). Predictor and moderator variables were standardized to minimize multicollinearity and improve interpretability. Moderation was examined using SEM to assess these interactions impact on mental health outcomes (see Figs. 2). Bootstrapping approach was also used to deal the multivariate normality potential violation issues.

## Results

### Mediation analyses

 The hypothesized SEM showed an acceptable fit to the data. Although the chi-square test was statistically significant (*χ²* =1686.14, *df* = 361, *p* < 0.001), which is common in large samples, other fit indices supported the adequacy of the model: the Comparative Fit Index (CFI) was 0.85, the Tucker-Lewis Index (TLI) was 0.83, the Root Mean Square Error of Approximation (RMSEA) was 0.07 (90% CI: 0.066, 0.073), and the Standardized Root Mean Square Residual (SRMR) was 0.05. These values indicate that the model reasonably represents the observed data.

 Regarding the correlations among the Big Five traits, Openness was significantly and positively associated to all other traits (at *p* < 0.01 and *p* < 0.001). Openness was significantly and positively correlated with Conscientiousness (*r* = 0.269, *p* < 0.001), Extraversion (*r* = 0.250, *p* < 0.001), Agreeableness (*r* = 0.100, *p* = 0.020), and Neuroticism (*r* = 0.103, *p* = 0.036). Conscientiousness was also significantly and positively related to all other traits (at *p* < 0.01 and *p* < 0.001), except for its relation to Neuroticism, which was not significant. Conscientiousness was significantly correlated with Extraversion (*r* = 0.352, *p* < 0.001) and Agreeableness (*r* = 0.147, *p* = 0.001), but not with Neuroticism (*r* = 0.032, *p* = 0.532). The correlations between Extraversion, Agreeableness, and Neuroticism were not significant. The correlations between Extraversion and Agreeableness (*r* = 0.048, *p* = 0.302), Extraversion and Neuroticism (*r* = 0.046, *p* = 0.345), and Agreeableness and Neuroticism (*r* = 0.047, *p* = 0.304) were not significant.

 Moreover, Confirmatory factor analysis supported the latent structure of the Health Problems construct, with strong loadings for Anxiety (0.923), Somatization (0.823), and Depression (0.708), and a moderate loading for Social Dysfunction (0.477). The latent construct of Imposter Syndrome was similarly well defined by its 20 items (λ = 0.46 to λ = 0.80), although item 1 (λ = 0.16, “I have often succeeded on a test or task even though I was afraid that I would not do well before I undertook the task”) and item 2 (λ = 0.23, “I can give the impression that I’m more competent than I really am”) showed weaker loadings.

In terms of direct effects on Health Problems, Neuroticism was a strong positive predictor (*β* = 0.335, *p* < 0.001). Extraversion was weakly negatively associated with Mental Problems (*β* = -0.090, *p* = 0.026), indicating a slight protective effect. Openness (*β* = -0.062, *p* = 0.088), Conscientiousness (*β* = 0.021, *p* = 0.570), and Agreeableness (*β* = -0.077, *p* = 0.059) were not statistically significant predictors of Health Problems. Full results are presented in Table 2. When predicting Imposter Syndrome, Neuroticism again emerged as a significant positive predictor (*β* = 0.468, *p* < 0.001), while Agreeableness showed a small but significant negative association (*β* = -0.109, *p* = 0.005). Openness (*β* = 0.02, *p* = 0.592), Conscientiousness (*β* = -0.015, *p* = 0.739), and Extraversion (*β* = -0.010, *p* = 0.797) did not significantly predict Imposter Syndrome. In turn, Imposter Syndrome significantly predicted Health Problems (*β* = 0.219, *p* < 0.001), independent of personality traits. For details see Table 1.

 Mediation analysis revealed that Imposter Syndrome significantly mediated the relationship between Neuroticism and Health Problems (indirect *β* = 0.102, *p* < 0.001), suggesting that individuals high in Neuroticism indirectly are more likely to experience poor mental health due to elevated Imposter Syndrome. A similar but weaker mediating effect was found for Agreeableness (indirect *β* = -0.024, *p* = 0.014), indicating that higher Agreeableness is linked to lower Imposter Syndrome and, consequentially, less Health Problems. Indirect effects of Openness, Conscientiousness, and Extraversion were not statistically significant (see Tables 2 and 3 for details). In summary, these findings suggest that Imposter Syndrome mediates the relationship between Neuroticism and mental health and, to a lesser extent, between Agreeableness and mental health. Neuroticism remains a strong direct and indirect predictor of mental health problems, while Agreeableness shows a modest protective indirect effect. These results highlight the importance of Imposter Syndrome as a psychological mechanism linking personality to mental health outcomes, albeit with small effect sizes. The mediation observed for Neuroticism was partial, while for Agreeableness, it may suggest a weaker, potentially full mediation effect.

**Table 1:** Regression paths for big five personality traits, imposter syndrome, and mental health problems.

Predictor	Dependent Variable	Estimate	SE	Z	*p*	Variance	Std. Estimation
Openness	Mental Health	-0.114	0.067	-1.704	0.088	-0.179	-0.062
Conscientiousness		0.023	0.040	0.568	0.570	0.036	0.021
Extraversion		-0.115	0.051	-2.277	0.023	-0.181	-0.090
Agreeableness		-0.081	0.043	-1.888	0.059	-0.127	-0.077
Neuroticism		0.396	0.051	7.785	0.000	0.620	0.335
Openness	Imposter Syndrome	0.031	0.058	0.536	0.592	0.057	0.020
Conscientiousness		-0.014	0.041	-0.334	0.739	-0.025	-0.015
Extraversion		-0.011	0.042	-0.257	0.797	-0.020	-0.010
Agreeableness		-0.098	0.033	-2.935	0.003	-0.179	-0.109
Neuroticism		0.474	0.044	10.761	0.000	0.866	0.468
Imposter Syndrome	Mental Health	0.256	0.058	4.432	0.000	0.219	0.219

**Table 2:** Indirect effects of personality traits on mental health problems through imposter syndrome.

Path	Estimate	SE	Z	*p*	Variance	Std. Estimation
Openness → Mental Health	0.008	0.015	0.517	0.605	0.012	0.004
Conscientiousness → Mental Health	-0.004	0.011	-0.329	0.742	-0.006	-0.003
Extraversion → Mental Health	-0.003	0.011	-0.253	0.801	-0.004	-0.002
Agreeableness → Mental Health	-0.025	0.010	-2.446	0.014	-0.039	-0.024
Neuroticism → Mental Health	0.121	0.027	4.479	0.000	0.190	0.102

**Table 3:** Covariances of personality traits with imposter syndrome.

Covariate Pair	Covariance	SE	Z	*P*	Variance	Std. Estimations
Imposter Syndrome ~ ~ O	0.010	0.009	1.145	0.252	0.018	0.051
Imposter Syndrome ~ ~ C	-0.004	0.016	-0.28	0.779	-0.008	-0.014
Imposter Syndrome ~ ~ E	0.002	0.012	0.146	0.884	0.003	0.006
Imposter Syndrome ~ ~ A	-0.029	0.014	-2.013	0.044**	-0.053	-0.087
Imposter Syndrome ~ ~ N	0.137	0.015	8.864	< 0.001**	0.251	0.464

Note: O = Openness, C = Conscientiousness, E = Extraversion, A = Agreeableness, N = Neuroticism. The P-value indicates the statistical significance of the relationship.

### Moderation analyses

The structural equation model testing moderation effects demonstrated acceptable fit to the data. The chi-square statistic was significant (*χ²* =1819.01, *df* = 471, *p* < 0.001), and additional fit indices indicated a reasonably good model fit: CFI = 0.86, TLI = 0.83, RMSEA = 0.06 (90% CI: 0.06, 0.065), and SRMR = 0.05. These values suggest that the hypothesized model provides a fair representation of the observed data.

Correlations between the Big Five personality traits were generally weak, ranging from *r* = 0.032 to *r* = 0.352 (at *p* < 0.01 and *p* < 0.001). Similarly, correlations between the Big Five traits and Imposter Syndrome were generally weak, ranging from *r* = -0.087 to *r* = 0.464. Openness (*r* = 0.051, *p* = 0.252), Conscientiousness (*r* = -0.014, *p* = 0.779), and Extraversion (*r* = 0.006, *p* = 0.884) showed no significant association with Imposter Syndrome. Agreeableness had a significant negative correlation (*r* = -0.087, *p* = 0.044), and Neuroticism showed a moderately strong positive correlation (*r* = 0.464, *p* < 0.001) (see Table 3).The latent variable Health Problems was well-represented by its four subcomponents: Anxiety (*λ* = 0.922), Somatization (*λ* = 0.824), Depression (*λ* = 0.708), and Social Dysfunction (*λ* = 0.476). Likewise, the latent variable Imposter Syndrome was reliably indicated by its 20 items (*λ* = 0.46 to *λ* = 0.80), except for item 1 (λ = 0.16, “I have often succeeded on a test or task even though I was afraid that I would not do well before I undertook the task”) and item 2 (λ = 0.23, “I can give the impression that I’m more competent than I really am”), which loaded weakly.

As expected, Neuroticism was a strong significant predictor of poorer health (*β* = 0.335, *p* < 0.001). Extraversion showed a small but statistically significant negative association with Health Problems (*β* = −0.094, *p* = 0.026), suggesting a slight protective effect. The other personality traits, Openness, Conscientiousness, and Agreeableness were not significantly associates to Health Problems. Imposter Syndrome showed a small positive association with Health Problems (*β* = 0.217, *p* < 0.001). Indicating that higher levels of imposter feelings were linked to worse mental health.

The results of the moderation analyses revealed that two interaction terms emerged as statistically significant. The interaction between Conscientiousness and Imposter Syndrome was significant and positively associated to Health Problems (*β* = 0.07, *p* < 0.001), while the interaction between Neuroticism and Imposter Syndrome was significant but negatively associated (*β* = − 0.04, *p* < 0.001). These effects were statistically reliable but weak in magnitude. No other interaction terms reached significance (see Table 4). Moreover, the correlations patterns between interaction terms and personality traits were weak, ranging from 0.001 to 0.281, indicating limited multicollinearity. Likewise, the correlations between interaction terms and Imposter Syndrome (ranging from − 0.005 to 0.089) and among interactions terms (ranging from − 0.051 to 0.281) were weak. However, the correlation between the interaction term Conscientiousness x Imposter Syndrome and Extraversion x Imposter Syndrome was stronger (*β* = 0.433, *p* < 0.001). Overall, these results suggest that Imposter Syndrome slightly modifies the influence of Conscientiousness and Neuroticism on mental health outcomes. However, the small size of these effects cautions against overinterpreting their practical impact. Moderation by Imposter Syndrome appears to be present, but subtle and trait specific.

**Table 4:** Covariance and correlation estimates for big five traits, imposter syndrome, and their interaction.

Covariate Pair	Covariance/ Variance/Estimate	SE	Z	*P*	Std. Estimate (*r*)
***Traits with Interaction Terms***					
Openness – O x IS Interaction	0.113	0.031	3.62	< 0.001***	0.281
Conscientiousness – O x IS Interaction	0.006	0.037	0.161	0.872	0.009
Extraversion – O x IS Interaction	0.011	0.029	0.378	0.705	0.019
Agreeableness – O x IS Interaction	-0.036	0.034	-1.032	0.302	-0.051
Neuroticism – O x IS Interaction	0.001	0.04	0.023	0.981	0.002
Openness – C x IS Interaction	0.004	0.022	0.161	0.872	0.008
Conscientiousness – C x IS Interaction	-0.022	0.065	-0.337	0.736	-0.029
Extraversion – C x IS Interaction	0.071	0.038	1.868	0.062	0.110
Agreeableness – C x IS Interaction	-0.081	0.038	-2.149	0.032*	-0.103
Neuroticism – C x IS Interaction	0.048	0.045	1.07	0.285	0.068
Openness – E x IS Interaction	0.008	0.021	0.379	0.705	0.019
Conscientiousness – E x IS Interaction	0.083	0.044	1.866	0.062	0.123
Extraversion – E x IS Interaction	0.007	0.046	0.159	0.873	0.013
Agreeableness – E x IS Interaction	-0.032	0.03	-1.048	0.294	-0.045
Neuroticism – E x IS Interaction	0.094	0.04	2.349	0.019*	0.149
Openness – A x IS Interaction	-0.02	0.02	-1.041	0.298	-0.050
Conscientiousness – A x IS Interaction	-0.077	0.036	-2.152	0.031*	-0.115
Extraversion – A x IS Interaction	-0.026	0.025	-1.055	0.291	-0.045
Agreeableness – A x IS Interaction	0.112	0.055	2.054	0.040*	0.159
Neuroticism – A x IS Interaction	-0.087	0.037	-2.352	0.019*	-0.139
Openness – N x IS Interaction	0.001	0.025	0.025	0.98	0.001
Conscientiousness – N x IS Interaction	0.051	0.045	1.147	0.252	0.068
Extraversion – N x IS Interaction	0.087	0.035	2.485	0.013*	0.134
Agreeableness – N x IS Interaction	-0.098	0.039	-2.482	0.013*	-0.124
Neuroticism – N x IS Interaction	-0.016	0.059	-0.273	0.785	-0.023
***Imposter Syndrome with Interaction Terms***					
Imposter Syndrome – O x IS Interaction	-0.003	0.047	-0.071	0.944	-0.005
Imposter Syndrome – C x IS Interaction	0.057	0.057	1.004	0.316	0.080
Imposter Syndrome – E x IS Interaction	0.055	0.048	1.156	0.248	0.086
Imposter Syndrome – A x IS Interaction	-0.05	0.049	-1.027	0.305	-0.080
Imposter Syndrome – N x IS Interaction	-0.008	0.056	-0.147	0.883	-0.012
***Inter-covariances of Interaction Terms***					
O x IS Interaction – C x IS Interaction	0.321	0.143	2.244	0.025*	0.215
O x IS Interaction – E x IS Interaction	0.302	0.126	2.392	0.017*	0.225
O x IS Interaction – A x IS Interaction	0.084	0.111	0.758	0.449	0.063
O x IS Interaction – N x IS Interaction	0.007	0.16	0.044	0.965	0.005
C x IS Interaction – E x IS Interaction	0.657	0.165	3.989	< 0.001***	0.433
C x IS Interaction – A x IS Interaction	0.291	0.133	2.189	0.029*	0.194
C x IS Interaction – N x IS Interaction	-0.226	0.173	-1.309	0.190	-0.134
E x IS Interaction – A x IS Interaction	0.135	0.100	1.356	0.175	0.100
E x IS Interaction – N x IS Interaction	-0.226	0.163	-1.382	0.167	-0.149
A x IS Interaction – N x IS Interaction	-0.360	0.136	-2.642	0.008**	-0.239

Note. SE = Standard Error; Std. Estimate (r) = Standardized Estimate (Correlation). O = Openness; C = Conscientiousness; E = Extraversion; A = Agreeableness; N = Neuroticism; IS = Imposter Syndrome. The “x IS Interaction” terms refer to the interaction between the specified standardized personality trait and Imposter Syndrome. * *p* < 0.05. ** *p* < 0.01. *** *p* < 0.001.

## Discussion

We aimed to clarify whether Imposter Syndrome operates primarily as a mechanism (mediator) or as a contextual amplifier (moderator) linking Big Five personality traits to mental health problems among Malaysian university students. By estimating parallel mediation and moderation models within a comparable framework yielded a differentiated but consistent picture. First, Neuroticism emerged as the most reliable correlate of both higher Imposter Syndrome and poorer mental health, in line with extensive evidence that emotional stability, self-criticism, and stress reactivity heighten internalizing risk^45–47,26,48–51^. Extraversion showed a modest protective association with mental health, which is compatible with the idea that social engagement may provide emotional resources^52,53^. Agreeableness related weakly and negatively to Imposter Syndrome, suggesting that other-oriented, cooperative and trusting individuals may be less prone to interpret achievement through a self-doubting, fraudulence lens^54–56^. In contrast, Openness, Conscientiousness, and Agreeableness showed limited relevance for predicting Imposter Syndrome. In addition, most traits, except Neuroticism, demonstrated minimal impact on mental health outcomes.

With regards to mediation, Imposter Syndrome partially accounted for the Neuroticism-mental health link and conveyed a very small pathway for Agreeableness. No indirect effects emerged for Openness, Conscientiousness, or Extraversion. Taken together, Imposter Syndrome appears to transmit some, but not most, of the risk caused by Neuroticism, and its mediating role is trait-specific rather than universal. In other words, while Neuroticism and Agreeableness may slightly influence imposter feelings that subsequently impact mental health, other traits may operate through different psychological mechanisms. This is in line with studies reporting mixed evidence for Imposter Syndrome as a mediator across constructs and samples (e.g., null mediation for perfectionism → depression in medical residents; and for self-esteem → career satisfaction^57^). Hence, suggesting that other mechanisms (e.g., perfectionistic concerns, emotion dysregulation, low self-acceptance) likely account for a larger share of personality–health covariance.

The moderation analyses indicated that Imposter Syndrome slightly altered personality traits-mental health associations. Specifically, higher Conscientiousness was associated with slightly poorer mental health at higher Imposter Syndrome, which is consistent with overcontrol and self-doubt when high standards meet imposter fears^22,58,59,24^. Moreover, the association between Neuroticism and poorer mental health was marginally stronger at higher levels of imposter feelings. which aligns with well-established research linking emotional instability to internalizing difficulties^60,46,61^. Nevertheless, as for mediation effects, both interaction effects were small and explained very little additional variance, so we suggest that they have limited practical meaning^62^. Their statistical detection may, for instance, reflect the large sample size rather than meaningful psychological processes.

In sum, neither the mediation nor the moderation indicates that Imposter Syndrome is a central pathway or boundary condition for the relationship between Big Five personality traits and mental health in our population of Malaysian university students. Statistically significant effects do appear, but their magnitudes are modest (see Ferguson^62^, who suggest effect sizes < 0.20 fall beneath the recommended threshold for practical significance in social science research). The most plausible interpretation of the results is therefore that Imposter Syndrome reflects a surface expression of broader liabilities, specially Neuroticism, with small incremental impact once those dispositions are accounted for. That being said, Malaysia’s collectivistic context, emphasizing humility/modesty, meeting family expectations, and avoiding “loss of face”, may shape both the experience and reporting of impostor feelings^63^. Students might downplay distress or self-doubt to align with social desirability, attenuating observed associations between Imposter Syndrome and other variables, contributing to the small effect sizes in the present study. Indeed, cross-cultural research shows variability in Imposter Syndrome levels and correlates across Asian vs. Western samples^45,64,65^ and even across Asian-heritage students in USA settings who often report higher Imposter Syndrome than their African American or Latino peers— a pattern attributed to cultural influences such as the “model minority” pressure and fear of not meeting high collective expectations^66,67^.

### Theoretical and practical implications

Our findings support a restrained view of Imposter Syndrome. Rather than a distinct, influential factor in academic dysfunction (e.g^22^.,, Imposter Syndrome may be best understood as an epiphenomenon, that is a surface expression of broader vulnerabilities such as Neuroticism, self-doubt, and perfectionistic standards; thus offering limited incremental value beyond those traits and the issues with emotional regulation thereof^68^. This interpretation of our results resonates with the jingle–jangle problem in psychology, where relabeled or overlapping constructs can create the illusion of novelty without added explanatory power^69^. 

Conceptually, the pattern fits with Trait Activation Theory (evaluation-laden contexts cue self-critical appraisals; Imposter Syndrome as a mechanism) and Person–Environment Fit Theory (mismatch intensifies strain, Imposter Syndrome as boundary condition)^36,37,35^. However, the small effects here imply that when these processes operate, they might do so weakly in student samples. Practically, this suggests for targeting root vulnerabilities rather than the impostor label itself. Interventions that bolster emotion regulation, self-compassion, adaptive coping, and that address perfectionistic concerns and negative self-evaluation, are likely to yield broader benefits than narrowly “anti-imposter” programs^68,70^. In Malaysian university settings, culturally responsive supports that normalize help-seeking, strengthen interpersonal trust, and reduce evaluation anxiety may be especially relevant^71,^^72,73^. In other words, by focusing on root vulnerabilities rather than surface labels^74^, universities can design interventions that are both culturally responsive and clinically meaningful.

Future research should continue to investigate culturally grounded psychological and environmental mechanisms that influence student well-being in diverse educational settings. In particular, studies might explore how cultural dimensions (e.g. individualism vs. collectivism) moderate the experience and impact of imposter feelings. Such work can inform the adaptation of theoretical constructs and interventions to better fit non-Western contexts, ultimately providing more valid and effective strategies for supporting student mental health across cultures. As other Western-developed constructs, Imposter Syndrome may require cultural adaptation to ensure conceptual relevance and explanatory power outside the Western context. Indeed, research has raised concerns that instruments such as the Clance Imposter Phenomenon Scale may not capture imposter feelings equivalently across cultures, given evidence of cross-cultural differences in measurement properties and response styles^75,76^.

### Limitations and future directions

The cross-sectional design of our study precludes any conclusions about causality. Longitudinal or cross-lagged studies are needed to test directionality among personality traits, Impostor Syndrome, and mental health problems over time. The study relied on self-report questionnaires, introducing the possibility of response biases such as social desirability, potentially amplified in collectivistic context where modesty shape responding. Also, the data were collected from a single university, which may limit the generalizability. To ensure the results are not culture-bound, it is important to validate these findings in diverse cultural settings. Future studies should replicate this research in different countries or populations, examining whether similar relationships among impostor feelings, personality traits, and mental health outcomes hold true elsewhere. Beyond simply replicating in other settings, future research should explore the role of cultural value systems (such as individualism vs. collectivism) in shaping the impostor experience. For example, cultural dimensions might moderate how strongly the Imposter Syndrome relates to personality and mental health. Investigating these cultural moderators could clarify why impostor feelings manifest differently across contexts and how culture might buffer or exacerbate their impact.

Although the sample included both Malaysian and international students, we did not conduct group comparisons here. This decision was based on the absence of significant differences in mental health outcomes between Malaysian and international students in the original studies using the same dataset. Nonetheless, future studies should conduct measurement invariance tests for Imposter Syndrome, then use multi-group SEM to test whether structural paths differ by nationality/culture. Moreover, given small effects, the next step is stronger causal designs, such as randomized interventions that (a) reduce impostor cognitions and/or (b) tune evaluative context, with pre-post changes in mental health; ecological momentary assessment to test situational activation (Trait Activation Theory); and non-linear analyses to detect moderation regions that simple slopes may miss. At the construct level, researchers can incorporate facet-level and maladaptive variants (e.g., perfectionistic concerns tied to Conscientiousness), and consider alternative models (e.g., network analysis; bifactor/parcel-free Imposter Syndrome measurement) to test whether different representations yield stronger or more specific pathways.

Additionally, some personality traits (especially Openness and Conscientiousness) did not show significant effects in our results. This absence of effect may reflect limitations in how these traits were measured rather than true lack of influence. There could be cultural mismatches in the scale items or unique characteristics of our sample that masked their role. Future research should consider assessing these traits with alternative psychometric tools or more nuanced models. For example, incorporating maladaptive variants of traits (such as Persistence, which is related to Conscientiousness), examining specific facets of broad traits, or using personality models that include both temperament and self-regulatory traits such as Cloninger’s biopsychosocial model of personality^9^ might reveal clearer links between personality, impostor feelings, and mental health.

## Conclusion

Taken together, our findings suggest that Imposter syndrome is not a distinct factor driving distress on its own, but rather a surface manifestation of broader personality tendencies, particularly high Neuroticism, that predispose individuals to mental health challenges. This perspective implies that interventions might be more effective if they target those core personality factors, for example, efforts to reduce emotional instability and build resilience^74^ could yield greater improvements in mental health than interventions focusing solely on alleviating impostor feelings.

We also encourage future research to examine additional psychological and environmental factors to clarify how personality traits influence mental health outcomes. Psychological characteristics such as self-efficacy^77–79^, emotion regulation^80^, perfectionism^81,82^, resilience^83,84^, and coping strategies^85,86^ are known to shape individual responses to stress and may operate as mediators or moderators. Likewise, environmental conditions, including climate^87^, supervisor and peer support^88,89^, cultural expectations^90^, and organizational demands or pressure^91^, could influence how underlying personality profiles manifest as mental health risks or protective outcomes. Identifying these psychological and contextual mechanisms will deepen our theoretical understanding and guide more targeted interventions in educational and occupational settings. Such a comprehensive approach can clarify how impostor feelings fit into the bigger picture of personality and well-being.

*Don’t let the noise of others’ opinions drown out your own inner voice*

— Steve Jobs.

## Data Availability

The datasets used and/or analyzed during the current study are available from the corresponding author upon reasonable request.
